
JOURNAL INFORMATION
==============================
NLM Title Abbreviation: Wirtschaftsdienst
Journal ID: Wirtschaftsdienst
NLM Journal ID: 101086612

Wirtschaftsdienst (Hamburg, Germany : 1949)

ISSN: 0043-6275

ARTICLE INFORMATION
==============================
PMCID: PMC8800427
PMID: 35125547
DOI: 10.1007/s10273-022-3086-7
Article ID: 3086
Article version: 1
Subjects: Zeitgespräch

Finanzierung von Zukunftsinvestitionen: Pragmatische Lösungen sind gefragt

Financing Investments in the Future: Pragmatic Solutions Are Needed

Schnitzer Monika Schnitzer@econ.lmu.de
1
Truger Achim achim.truger@uni-due.de
2
1 grid.5252.0 0000 0004 1936 973X Seminar f. Komparative Wirtschaftsforschung, Ludwig-Maximilians-Universität München, Akademiestr. 1, 80799 München, Deutschland
2 grid.5718.b 0000 0001 2187 5445 Institut für Sozioökonomie, Universität Duisburg-Essen, Lotharstr. 65, 47057 Duisburg, Deutschland
Electronic publication date: 2022 Jan 29
Print publication date: 2022
Volume: 102
Issue: 1
First page: 11
Last page: 14
Copyright: © Der/die Autor:in 2022
License: Open Access: Dieser Artikel wird unter der Creative Commons Namensnennung 4.0 International Lizenz veröffentlicht (https://creativecommons.org/licenses/by/4.0/deed.de).
Open Access wird durch die ZBW — Leibniz-Informationszentrum Wirtschaft gefördert.
License URL: https://creativecommons.org/licenses/by/4.0/

Keywords: E62, H54, H62
issue-copyright-statement: © The Author(s) 2022

==============================
Since the new German federal government has imposed tight fiscal policy restrictions on itself, a pragmatic approach is needed to finance the spending requirements of the transformation. The financing instruments mentioned in the coalition agreement appears to be suitable in principle for meeting key fiscal challenges facing the federal government in the coming years. However, they need to be quantified in concrete terms as quickly as possible and designed to ensure legal security in order to send reliable signals for the necessary capacity building, particularly in the construction industry and public administration.

Prof. Dr. Monika Schnitzer ist Inhaberin des Lehrstuhls für Komparative Wirtschaftsforschung an der Ludwig-Maximilians-Universität München und Mitglied des Sachverständigenrates zur Begutachtung der gesamtwirtschaftlichen Entwicklung.

Prof. Dr. Achim Truger ist Professor für Sozioökonomie mit dem Schwerpunkt Staatstätigkeit und Staatsfinanzen an der Universität Duisburg-Essen und Mitglied des Sachverständigenrates zur Begutachtung der gesamtwirtschaftlichen Entwicklung.

Die bereits seit geraumer Zeit lebhaft geführte Debatte um Zukunftsinvestitionen und ihre Finanzierung dauert auch nach Präsentation des Koalitionsvertrags der Ampelkoalition und ihres Entwurfs für den Nachtragshaushalt 2021 an. Mindestens drei Punkte sind dabei umstritten: *Erstens* wird diskutiert, wie hoch die Investitionsbedarfe überhaupt sind. *Zweitens* geht es um die Finanzierung der Bedarfe und zwar vor allem darum, ob sie aus ökonomischer Sicht eine höhere Nettokreditaufnahme rechtfertigen können. *Drittens* ist umstritten, welche Spielräume im Rahmen der Schuldenbremse für kreditfinanzierte Investitionen bestehen und ob der Koalitionsvertrag und der Nachtragshaushalt diese in ausreichender und rechtlich zulässiger Weise nutzen.

## Massive Finanzbedarfe nicht zu leugnen

Trotz zahlreicher einschlägiger Studien wird gelegentlich argumentiert, die öffentlichen Investitionsbedarfe ließen sich eigentlich gar nicht seriös quantifizieren. So sei die Abgrenzung der Investitionen von konsumtiven Ausgaben schwierig, manche der vorgelegten Schätzwerte seien aufgrund der Beteiligung von Interessengruppen wahrscheinlich bewusst zu hoch angesetzt. Zudem hänge der öffentliche Investitionsbedarf stark von staatlichen Rahmensetzungen, insbesondere der Höhe des $CO_2$-Preises, ab, da ein hinreichend hoher $CO_2$-Preis für die Finanzierung ausreichender Investitionen durch private Investor:innen sorgen würde (Feld et al., 2021a und 2021b).

Ganz so unübersichtlich ist die Lage allerdings nicht. Natürlich lassen sich Bedarfe nie ohne Fehlermarge quantifizieren. Ungefähre Größenordnungen lassen sich jedoch durchaus plausibilisieren. So hat sich der Sachverständigenrat zur Begutachtung der gesamtwirtschaftlichen Entwicklung (SVR) für zahlreiche Maßnahmen ausgesprochen, die zu einem erheblichen Ausgabenbedarf führen. Allein die Energiepreisreform, also die Absenkung der Steuer- und Abgabenbelastung auf elektrischen Strom zur Förderung der Sektorkopplung, würde übergangsweise zusätzlich mit bis zu 20 Mrd. Euro jährlich zu Buche schlagen (SVR, 2020, Ziff. 391 ff.). Erhebliche Bedarfe werden außerdem in der Digitalisierung, der Infrastruktur, der öffentlichen Verwaltung sowie der Bildung identifiziert (SVR, 2019, 2020 und 2021). So wären beispielsweise ergänzende Maßnahmen für den Ausbau von Infrastruktur und öffentlichem Verkehrssystem nötig, selbst wenn der $CO_2$-Preis wünschenswerterweise tatsächlich zum Leitinstrument der Klimapolitik avancieren würde. Zudem liegt bisher kein Konzept vor, wie ohne staatliche Unterstützung die drohenden internationalen Wettbewerbsnachteile der deutschen Industrie durch einen hohen $CO_2$-Preis verhindert werden oder sozial benachteiligte Haushalte sich an den höheren $CO_2$-Preis anpassen sollen. Die dafür notwendigen Subventionen und Transfers gelten zwar nicht als Investitionen. Transformationsbedingt sind sie dennoch und finanziert werden müssen sie gleichwohl.

Anstatt die Bedarfe kleinzureden, muss eine finanzpolitische Strategie für die Transformation daher eine überzeugende Finanzierungsstrategie für jährliche Ausgaben in mittlerer zweistelliger Milliardenhöhe aufzeigen (Bardt et al., 2019; Wissenschaftlicher Beirat beim Bundesministerium für Wirtschaft und Energie, 2020; Krebs und Steitz, 2021; Dullien et al., 2022).

**Prof. Dr. Monika Schnitzer** ist Inhaberin des Lehrstuhls für Komparative Wirtschaftsforschung an der Ludwig-Maximilians-Universität München und Mitglied des Sachverständigenrates zur Begutachtung der gesamtwirtschaftlichen Entwicklung.

**Prof. Dr. Achim Truger** ist Professor für Sozioökonomie mit dem Schwerpunkt Staatstätigkeit und Staatsfinanzen an der Universität Duisburg-Essen und Mitglied des Sachverständigenrates zur Begutachtung der gesamtwirtschaftlichen Entwicklung.

* Der Beitrag lehnt sich stark an unsere gemeinsame Position zum Thema „Mobilisierung von Investitionen und ihre Finanzierung" im letzten Jahresgutachten des Sachverständigenrates an (SVR, 2021, Ziff. 229-245), wurde jedoch vor dem Hintergrund des Koalitionsvertrags und des Nachtragshaushalts aktualisiert und ergänzt.

### Höhere Nettokreditaufnahme ökonomisch gut zu rechtfertigen

Eine Kreditfinanzierung zukunftsbezogener öffentlicher Ausgaben lässt sich aus ökonomischer Sicht grundsätzlich gut rechtfertigen. So legt die Verwirklichung des intertemporalen Äquivalenzprinzips es nahe, öffentliche Nettoinvestitionen aus Gründen der Generationengerechtigkeit durch Kreditaufnahme zu finanzieren (Musgrave, 1959; SVR, 2007; Truger, 2015). Nettoinvestitionen erhöhen den Kapitalstock und stiften Nutzen für zukünftige Generationen. Daher sollten letztere über den Schuldendienst auch zur Finanzierung beitragen. Zukünftige Generationen erben dann zwar eine öffentliche Schuldenlast, sie erhalten dafür jedoch einen erhöhten Kapitalstock. Ein Verzicht auf Kreditfinanzierung würde wegen der dann notwendig werdenden Steuererhöhungen oder Ausgabenkürzungen einseitig die heutige Generation belasten und dieser zum Nachteil zukünftiger Generationen zu geringe Anreize für öffentliche Investitionen bieten. Dieses Anreizproblem verschärft sich unter dem Druck zur Haushaltskonsolidierung, weil eine Kürzung der disponiblen öffentlichen Investitionen häufig als einfachste Möglichkeit zur Verringerung des Haushaltsdefizits erscheint (Barbiero und Darvas, 2014). Im Rahmen einer verlässlichen langfristigen öffentlichen Investitionsstrategie spricht daher viel für eine Verstetigung öffentlicher Investitionen, auch durch Kreditfinanzierung. Dadurch lässt sich gleichzeitig ein glaubwürdiges Signal für den Aufbau von Kapazitäten in Bauwirtschaft und Planungsämtern und damit den Abbau einiger der zweifelsohne bestehenden nicht finanziellen Hemmnisse für öffentliche Investitionen (Wissenschaftlicher Beirat beim Bundesministerium für Wirtschaft und Energie, 2020) senden.

### Koalitionsvertrag und Nachtragshaushalt: Erhebliche potenzielle Spielräume

Die neu angetretene Ampelkoalition hat sich beim angestrebten Normalisierungskurs nach der Corona-Krise und bei der Finanzierung der Transformation sehr enge Grenzen gesetzt. Steuererhöhungen sollen grundsätzlich ausgeschlossen sein. Gleichzeitig will sie die grundgesetzliche Schuldenbremse nicht reformieren, aber schon im Jahr 2023 zur Regelgrenze von 0,35 % des BIP für die strukturelle Nettokreditaufnahme zurückkehren (SPD, Bündnis90/Die Grünen, FDP, 2021). Somit bleiben der Koalition zur Schaffung von Finanzierungsspielräumen nur Kürzungen auf der Ausgabenseite oder die Nutzung von Kreditfinanzierungsmöglichkeiten im Rahmen der Schuldenbremse.

Die Steuerschätzung vom November 2021 (BMF, 2021) lässt gegenüber der letzten Steuerschätzung vom Mai 2021 spürbare Mehreinnahmen von strukturell 12 Mrd. Euro jährlich über die gesamte Legislaturperiode von 2022 bis 2025 erwarten. Auch wenn sich dadurch nach aktueller Schätzung die finanzpolitische Situation etwas entspannt, würde dieser Spielraum nur einen Teil des benötigten Finanzbedarfs decken, ganz abgesehen davon, dass damit zu rechnen ist, dass die wirtschaftliche Erholung sich angesichts der Omikron-Variante weiter verschieben wird.

Eine Überprüfung der Staatsausgaben hinsichtlich der Effizienz der Mittelverwendung und der Vermeidung der Verschwendung von Steuergeldern sollte selbstverständlich sein und mag mittel- bis langfristig ausgabenseitige Spielräume eröffnen. Es ist aber unwahrscheinlich, dass sich allein dadurch zweistellige Milliardenbeträge einsparen lassen. Größere Spielräume ließen sich nur durch eine erheblich veränderte finanz- und sozialpolitische Schwerpunktsetzung erreichen. Dies erfordert jedoch eine ausführliche Analyse und Diskussion, die sicher auch nicht kurzfristig zur Freistellung von Mitteln führen würde. Dies gilt auch für den Abbau klimapolitisch kontraproduktiver Subventionen, der mittel- bis langfristig durchaus eine wichtige Rolle spielen könnte (Beermann et al., 2021). Im Koalitionsvertrag findet sich über generelle Überprüfungsabsichten hinaus wenig Konkretes. Genannt werden nur eine verminderte Förderung von Plug-in-Hybriden, sowie die tatsächliche Erhebung einer Plastikabgabe mit insgesamt vermutlich sehr geringem Finanzvolumen. Die Umsetzung der EU-Energiesteuerrichtlinie, die die steuerliche Angleichung von Dieselkraftstoff und Benzin vorsieht, dürfte ebenfalls kaum Finanzspielräume eröffnen, da bereits eine Kompensation der Dieselfahrenden über die Kfz-Steuer ins Spiel gebracht wird.

Daher wird man nicht um eine pragmatische Mobilisierung von Verschuldungsspielräumen im Rahmen der grundgesetzlichen Schuldenbremse herumkommen. Hierfür kommen zunächst Maßnahmen infrage, die eher temporäre Spielräume ermöglichen. Laut Koalitionsvertrag sollen etwa, ähnlich wie auch vom SVR (2021, Ziff. 151 ff.) vorgeschlagen, die Tilgungspläne für die Corona-Kredite zusammengefasst und die Tilgung von 20 auf 30 Jahre gestreckt und erst im Jahr 2028 begonnen werden. Für die gesamte Legislaturperiode entsteht dadurch allerdings lediglich ein Spielraum von gut 6 Mrd. Euro. Denn die wesentlichen Tilgungen sollten auch nach bisheriger Planung erst nach Ende der Legislaturperiode einsetzen. Als zweite Möglichkeit könnte eine modifizierte Schätzung des Produktionspotenzials im Rahmen der Konjunkturbereinigung wenigstens in der Erholungsphase einige Spielräume eröffnen und in Zukunft eine prozyklische Finanzpolitik vermeiden helfen (Heimberger und Truger, 2020; Truger, 2020). Krebs et al. (2021) weisen darüber hinaus darauf hin, dass eine systematische Berücksichtigung wachstums- und potenzialsteigernder wirtschaftspolitischer Maßnahmen, wie etwa eine Ausweitung öffentlicher Investitionen, in der Steuer- und Potenzialschätzung allgemeine Spielräume für den Bund in der Größenordnung von 10 Mrd. Euro jährlich ab dem Jahr 2023 einbringen könnte. Ob es zu solchen Anpassungen kommen wird, ist offen; im Koalitionsvertrag ist lediglich von einer Überprüfung der Methode der Potenzialberechnung die Rede.

Grundsätzlich könnte auch die vom SVR (2020, 140 f.) in die Diskussion gebrachte Option einer schrittweisen Rückkehr zur Regelobergrenze für die strukturelle Neuverschuldung von 0,35 % des BIP in der Übergangsphase Haushaltsspielräume ermöglichen. Dafür müsste über 2022 hinaus die Ausnahmeregel der Schuldenbremse in Anspruch genommen werden. Möglich wäre dies, wenn die außergewöhnliche Notsituation die staatliche Finanzlage weiterhin erheblich beeinträchtigt, also ein Kausalzusammenhang zwischen der Corona-Krise und resultierenden erheblichen finanziellen Belastungen hergestellt werden kann (Korioth, 2020). In dem Maße, in dem noch coronabedingte Zusatzausgaben, etwa im Gesundheitsbereich oder zur Konjunkturstabilisierung, erforderlich sind oder fortwirken oder Mindereinnahmen bei Steuern oder Sozialversicherungsbeiträgen kompensiert werden müssten, sollte eine längere Inanspruchnahme der Ausnahmeregel rechtlich möglich sein. Dies gilt angesichts der konjunkturellen und finanziellen Belastungen durch die vierte Corona-Welle und die erheblichen Risiken der Omikron-Variante aktuell umso mehr. Der Koalitionsvertrag zieht diese Möglichkeit aber nicht in Betracht, sondern spricht sich für die Rückkehr zur Regelgrenze der Schuldenbremse im Jahr 2023 aus.

Für die Kreditfinanzierung von Investitionen kommen zwei Möglichkeiten in Betracht. Zum einen die Nutzung rechtlich selbständiger Extrahaushalte als Investitionsgesellschaften, die außerhalb der Schuldenbremse Kredite aufnehmen. Zum anderen die kreditfinanzierte Bildung oder Befüllung von Rücklagen oder rechtlich unselbständigen Sondervermögen, aus denen in späteren Jahren die notwendigen Ausgaben finanziert werden.

Rechtlich selbständige Extrahaushalte, deren Kreditaufnahme nicht von der Schuldenbremse erfasst wird, können privatrechtlich verfasste Unternehmen in öffentlichem Besitz oder Anstalten öffentlichen Rechts sein (Hermes et al., 2020). Im Rahmen der Schuldenbremse könnten damit neben schon bestehenden öffentlichen Unternehmen wie der Deutschen Bahn oder Körperschaften wie beispielsweise der Bundesanstalt für Immobilienaufgaben (BImA) auch andere öffentliche Investitionsgesellschaften gegründet werden, die auf konkrete Aufgaben fokussiert sind und durch die Bündelung von Expertise Synergien und Skaleneffekte nutzen (Wissenschaftlicher Beirat beim Bundesministerium für Wirtschaft und Energie, 2020). Wenn solche Gesellschaften einer klar definierten Sachaufgabe dienen und durch Bundesgesetz eingerichtet werden, könnten sie auch mit einer Kreditermächtigung ausgestattet werden (Hermes et al., 2020, 21 ff.). Die parlamentarische Kontrolle müsste über das Einrichtungsgesetz gewährleistet werden (Hermes et al., 2020, 30 ff.). Eine Bundesbürgschaft könnte sicherstellen, dass die Gesellschaft günstige Kreditkonditionen erhält. Im Koalitionsvertrag werden letztlich all diese Optionen angesprochen, wobei explizit die Deutsche Bahn und die BImA genannt und die besondere Rolle der Kreditanstalt für Wiederaufbau (KfW) bei der Unterstützung privater Investitionen betont werden. Konkrete Zahlen werden leider nicht genannt, allerdings zeigen Krebs et al. (2021), dass dadurch grundsätzlich erhebliche Finanzierungbeiträge aufgebracht werden können.

Auch Rücklagen oder rechtlich unselbständige Sondervermögen könnten Investitionsspielräume eröffnen. So will die Ampelkoalition im Nachtragshaushalt 2021 60 Mrd. Euro an nicht genutzten Kreditermächtigungen aus der Corona-Bekämpfung im Jahr 2021 in den zum Klima- und Transformationsfonds (KTF) umbenannten Energie- und Klimafonds einstellen und in den kommenden Jahren für Klimaschutzausgaben einsetzen (Deutscher Bundestag, 2021). Ob und unter welchen Bedingungen dies rechtlich zulässig ist, ist umstritten. Die Unionsfraktion hat bereits eine Verfassungsklage in Aussicht gestellt. Wie das Urteil des Staatsgerichtshofs des Landes Hessen (2021) gezeigt hat, ist eine sorgfältige verfassungsrechtlich tragfähige Begründung von großer Bedeutung. Auf jeden Fall muss die zur Befüllung notwendige Kreditaufnahme wohl ihrerseits über die Ausnahmeregel der Schuldenbremse gerechtfertigt werden, d. h. es muss ein hinreichender kausaler Zusammenhang zwischen der Corona-Krise und den aus dem KTF zu finanzierenden Maßnahmen hergestellt werden. Der Gesetzentwurf stellt den Zusammenhang dem Grunde nach über die Notwendigkeit der konjunkturellen Stabilisierung und die Kompensation coronabedingter Investitionslücken grundsätzlich plausibel dar. Eine genauere Spezifikation, warum und in welcher Höhe die zu finanzierenden Ausgaben zur Krisenbekämpfung notwendig sind, dürfte die Verfassungsfestigkeit des Nachtragshaushalts vermutlich noch erhöhen. Gelänge die Zuführung an den KTF, würde dies die Finanzierung der transformativen Ausgaben in den kommenden Jahren deutlich erleichtern. Sollte die Zuführung an den KTF als verfassungswidrig eingestuft werden, könnte alternativ eine längere Inanspruchnahme der Ausnahmeregel der Schuldenbremse für zur nachhaltigen Überwindung der Corona-Krise notwendige Ausgaben oder eine Ausgestaltung der relevanten Teile des KTF als rechtlich selbstständige Investitionsgesellschaft erwogen werden.

## Ausblick

Trotz der durch die Ampelkoalition gesetzten engen finanzpolitischen Restriktionen erscheinen die im Koalitionsvertrag genannten Finanzierungsinstrumente grundsätzlich als geeignet, wesentliche finanzpolitische Herausforderungen des Bundes durch die Transformation in den nächsten Jahren pragmatisch anzugehen. Allerdings bedarf es einer möglichst zügigen konkreten Quantifizierung und rechtssicheren Ausgestaltung, auch um verlässliche Signale für den notwendigen Kapazitätsaufbau insbesondere in der Bauwirtschaft und der öffentlichen Verwaltung zu setzen. Potenzielle rechtliche Risiken des Nachtragshaushalts können noch im Gesetzgebungsverfahren durch weitere Erläuterungen und konkretere Begründungen minimiert werden.



Literatur

Beermann, A.-C., S. Fiedler, M. Runkel, I. Schrems und F. Zerzawy (2021), Zehn klimaschädliche Subventionen sozial gerecht abbauen — ein Zeitplan. Eine Studie des Forums Ökologisch-Soziale Marktwirtschaft im Auftrag von Greenpeace.
Barbiero, F. und Z. Darvas (2014), In Sickness and in Health: Protecting and Supporting Public Investment in Europe.
Bardt, H., S. Dullien, M. Hüther und K. Rietzler (2019), Für eine solide Finanzpolitik. Investitionen ermöglichen!
BMF (2021), Scholz: „Steuerschätzung bestätigt unseren Kurs und macht Mut für die Zukunft“. Ergebnisse der 161. Steuerschätzung.
Deutscher Bundestag (2021), Gesetzentwurf der Bundesregierung. Entwurf eines Gesetzes über die Feststellung eines Zweiten Nachtrags zum Bundeshaushaltsplan für das Haushaltsjahr 2021 (Zweites Nachtragshaushaltsgesetz 2021), 13.12.2021, Bundestagsdrucksache 20/300.
Dullien, S., K. Rietzler und A. Truger (2022), Die Corona-Krise und die sozial-ökologische Transformation: Herausforderungen für die Finanzpolitik, WSI Mitteilungen, erscheint demnächst.
Feld L P Grimm V Reuter W H Zukunftsperspektiven sichern durch Reformen, nicht durch Schulden Wirtschaftsdienst 2021 101 6 418 424 10.1007/s10273-021-2935-0
Feld, L. P., V. Grimm und V. Wieland (2021b), Genug Spielraum. Klimaschutz geht in Deutschland auch ohne eine Lockerung der staatlichen Schuldenbremse, Frankfurter Allgemeine Zeitung, 14. November, 21.
Heimberger, P. und A. Truger (2020), Der Outputlücken-Nonsense gefährdet Deutschlands Erholung von der Corona-Krise, https://makronom.de/der-outputluecken-nonsense-gefaehrdet-deutschlands-erholung-von-der-corona-krise-36125 (2. Januar 2022).
Hermes, G., L. Vorwerk und T. Beckers (2020), Die Schuldenbremse des Bundes und die Möglichkeit der Kreditfinanzierung von Investitionen. Rechtslage, ökonomische Beurteilung und Handlungsempfehlungen.
Korioth, S. (2020), Die Reichweite notlagenbedingter struktureller Nettokreditaufnahme nach der Bremischen Landesverfassung (Art. 131a Abs. 3 BremLV) und die Bedeutung des „begründeten Ausnahmefalls“ nach dem Sanierungshilfengesetz (§ 2 Abs. 3 S. 2, Abs. 4 S. 2 SanG) angesichts der COVID-19-Pandemie Rechtsgutachtliche Stellungnahme.
Krebs, T. und J. Steitz (2021), Oeffentliche Finanzbedarfe fuer Klimainvestitionen im Zeitraum 2021–2030, https://ideas.repec.org/p/agz/wpaper/2103.html (2. Januar 2022).
Krebs, T., J. Steitz und P. Graichen (2021), Öffentliche Finanzierung von Klima- und anderen Zukunftsinvestitionen.
Musgrave, R. A. (1959), The theory of public finance. A study in public finance.
SVR (2007), Staatsverschuldung wirksam begrenzen. Expertise im Auftrag des Bundesministers für Wirtschaft und Technologie.
SVR (2019), Aufbruch zu einer neuen Klimapolitik, Sondergutachten.
SVR (2020), Corona-Krise gemeinsam bewältigen, Resilienz und Wachstum stärken.
SVR (2021), Transformation gestalten: Bildung, Digitalisierung und Nachhaltigkeit, Jahresgutachten 2021/22.
SPD, Bündnis90/Die Grünen, FDP (2021), Mehr Fortschritt wagen. Bündnis für Freiheit, Gerechtigkeit und Nachhaltigkeit, https://www.spd.de/fileadmin/Dokumente/Koalitionsvertrag/Koalitionsvertrag_2021–2025.pdf (2. Januar 2022).
Staatsgerichtshof des Landes Hessen (2021), Urteil Im Namen des Volkes in dem Verfahren zur Prüfung der Verfassungsmäßigkeit des Gesetzes über das Sondervermögen „Hessens gute Zukunft sichern“ (Gute-Zukunft-Sicherungsgesetz) vom 4. Juli 2020 (GVBl, 482), § 1 des Gesetzes über die Feststellung des Haushaltsplans des Landes Hessen für das Haushaltsjahr 2020 (Haushaltsgesetz 2020) vom 19. Februar 2020 (GVBl, 135) in der Fassung des Zweiten Gesetzes zur Änderung des Haushaltsgesetzes 2020 vom 4. Juli 2020 (GVBl, 485) in Verbindung mit dem Einzelplan 17, Kapitel 1701, Titel 234 01, 359 04 und 971 01, Kapitel 1703, Titel 334 04 und 883 08 des Haushaltsplans als Anlage zu diesem Gesetz sowie § 2 Absatz 12 Nummer 2 und Nummer 3 Buchstabe a, § 15a Absatz 4 und § 17 Satz 3 dieses Gesetzes sowie des Zweiten Gesetzes zur Änderung des Artikel 141-Gesetzes vom 2. Juli 2020 (GVBl, 472)
Truger, A. (2015), Implementing the Golden Rule for public investment in Europe. Safeguarding public investment and supporting the recovery.
Truger A Reforming EU Fiscal Rules: More Leeway, Investment Orientation and Democratic Coordination Intereconomics 2020 55 5 277 281 10.1007/s10272-020-0915-z
Wissenschaftlicher Beirat beim Bundesministerium für Wirtschaft und Energie (2020), Öffentliche Infrastruktur in Deutschland: Probleme und Reformbedarf.
